# Suppressing Charge Recombination in DSSCs with ZrO_2_ Compact Layers: Experimental Validation and Mathematical Modeling

**DOI:** 10.3390/nano16181192

**Published:** 2026-09-21

**Authors:** Halil İbrahim Yavuz, Macit Ozenbas

**Affiliations:** 1 Materials Science and Nanotechnology Engineering, Faculty of Engineering and Natural Sciences, Yeditepe University, 34785 Istanbul, Türkiye; 2 Metallurgical and Materials Engineering, Faculty of Engineering, Middle East Technical University, 06800 Ankara, Türkiye; ozenbas@metu.edu.tr

**Keywords:** dye-sensitized solar cell (DSSC), zirconium dioxide (ZrO_2_), electron blocking layer (EBL), quantum tunneling, recombination kinetics, mathematical modeling, electro-chemical impedance spectroscopy, sustainability

## Abstract

Since their breakthrough development, dye-sensitized solar cells (DSSCs) have emerged as highly promising third-generation photovoltaics; however, interfacial charge carrier recombination remains a persistent bottleneck. This study investigates the application of a wide bandgap ZrO_2_ electron blocking layer (EBL) to mitigate this recombination. A 48 nm ZrO_2_ EBL was deposited on fluorine-doped tin oxide (FTO) via hydrothermal treatment. Empirical results demonstrate a remarkable 43.9% enhancement in overall power conversion efficiency (6.77%) and a 69% improvement in total Incident Photon-to-Current Efficiency (IPCE) compared to bare FTO architectures. To theoretically validate the role of an insulating material as an EBL, the empirical data is supported by mathematical modeling, specifically utilizing Wentzel–Kramers–Brillouin (WKB) quantum tunneling probabilities and 1D diffusion–recombination kinetics. The models confirm that at nanoscale thicknesses, ZrO_2_ acts as a selective physical barrier that shifts the surface Fermi level and extends the electron lifetime to 0.0146 s. Furthermore, potential optimization pathways utilizing machine learning algorithms for ideal thickness prediction and IPCE clustering are discussed, paving the way for next-generation predictive device engineering. Ultimately, this dual empirical–theoretical approach provides a comprehensive understanding of the interfacial charge transport mechanisms, establishing a robust framework for designing highly efficient, leak-free photoanode architectures in next-generation photovoltaics.

## 1. Introduction

Since their breakthrough development by Grätzel and O’Regan in 1991, dye-sensitized solar cells (DSSCs) have established themselves as one of the most promising third-generation photovoltaic technologies. Compared to conventional silicon-based p-n junction devices, DSSCs offer highly attractive features: they require low-energy and cost-effective manufacturing processes, utilize non-toxic materials, and provide excellent performance under low-light or indoor environments [1,2]. Furthermore, their semi-transparency and color tunability make them ideal candidates for building-integrated photovoltaics (BIPV) and next-generation flexible electronic systems [3]. The fundamental operation of a DSSC relies on a continuous kinetic cycle. Photosensitizing dye molecules absorb incident photons and enter an excited state. These excited electrons are subsequently injected into the conduction band of a mesoporous wide-bandgap semiconductor typically TiO_2_, ZnO, or SnO_2_ which is deposited onto a transparent conductive oxide (TCO) substrate, such as fluorine-doped tin oxide (FTO) [4,5]. The injected electrons percolate through the nanoparticle network to the external circuit, while the oxidized dye is continuously regenerated by a redox mediator in the electrolyte [6]. Despite recent advancements pushing the limits of DSSC performance, a persistent bottleneck in achieving higher power conversion efficiency (η) remains interfacial charge carrier recombination [7]. Because the mesoporous TiO_2_ layer is inherently highly porous, regions of the bare TCO substrate remain exposed directly to the electrolyte. This direct contact induces a “short-circuit” effect, allowing for the backward transfer (leakage) of photogenerated electrons from the FTO back into the redox electrolyte, thus severely limiting the cell’s open-circuit voltage (Voc) and overall conversion efficiency [8,9].

**Figure 1:**
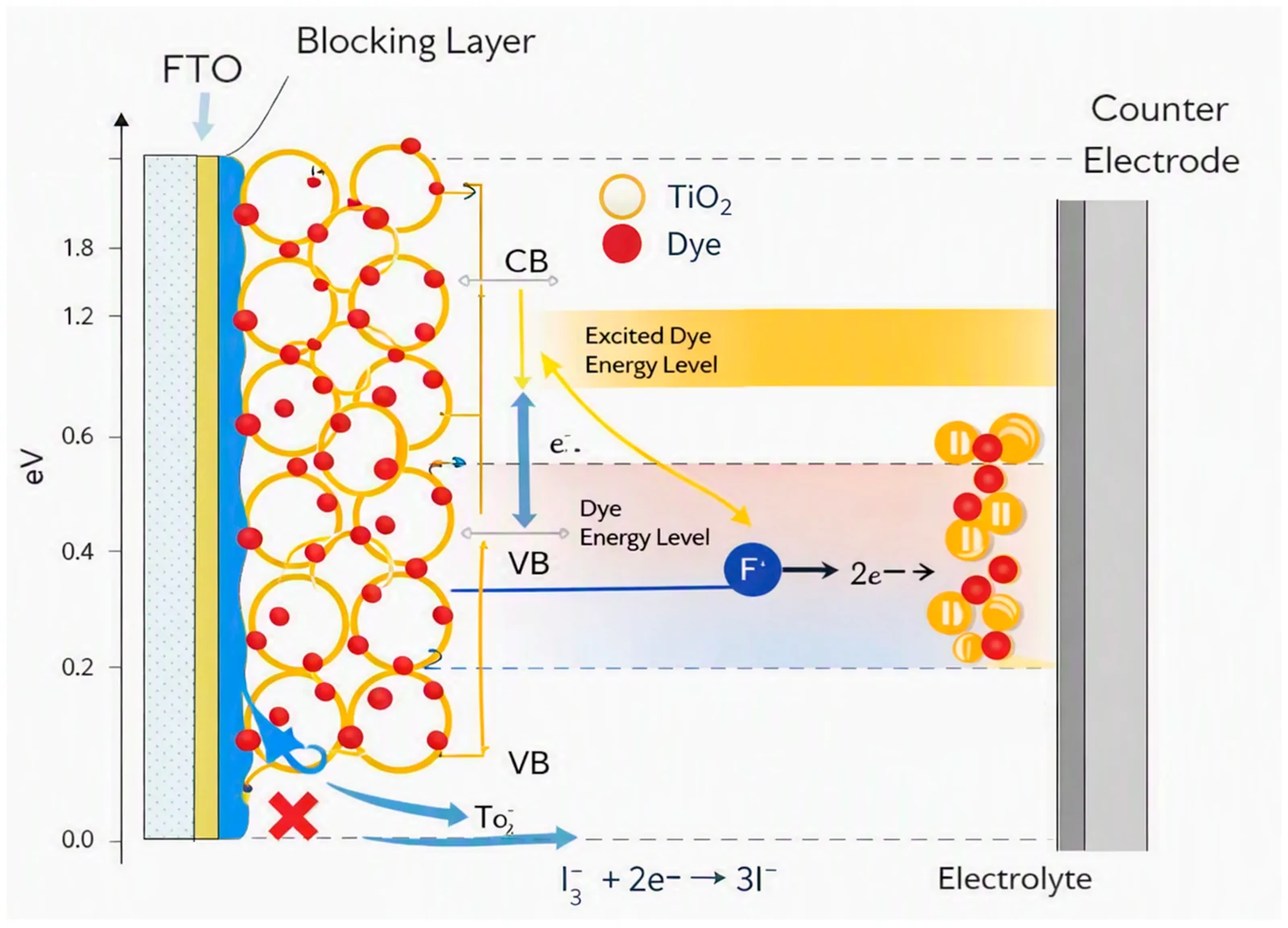
Schematic illustration of the blocking layer mechanism in a dye-sensitized solar cell (DSSC).

To suppress this undesirable reverse electron motion, the integration of an Electron Blocking Layer (EBL)—also known as a compact layer—between the FTO and the mesoporous semiconductor has become a critical device engineering strategy (Figure 1) [10]. An ideal EBL must fully cover the conductive substrate to isolate it from the electrolyte, while remaining thin enough to permit efficient forward electron conduction to the FTO [11]. Traditionally, a dense TiO_2_ thin film prepared via TiCl_4_ hydro-thermal treatment or spray pyrolysis is employed as the EBL [12,13]. However, depositing a dense TiO_2_ layer directly on the TCO often reduces optical transparency in the visible spectrum, thereby limiting the photon flux reaching the dye molecules. Consequently, recent research has pivoted toward wider band-gap metal oxides—such as Al_2_O_3_, ZnO, and SnO_2_—to formulate multi-functional heterojunction compact layers [14,15]. While ZrO_2_ has seen application as an insulating barrier in perovskite solar cells, to the best of our knowledge, its potential as a highly transparent EBL deposited directly onto FTO for liquid-state DSSCs has not been systematically reported. ZrO_2_ possesses a wide band gap, excellent thermal stability, and robust chemical inertness, making it a theoretically ideal candidate to block electron leakage without sacrificing the transparency of the photoanode. In this work, the EBL performance of ZrO_2_ in DSSCs is realized and thoroughly analyzed for the first time. We establish the optimal hydrothermal experimental conditions required to produce a highly transparent ZrO_2_ EBL that effectively minimizes electron leakage while maintaining a low charge carrier resistance. The optical and electronic characteristics at the interfacial region between the mesoporous TiO_2_ and the ZrO_2_ EBL are investigated using UV-Vis spectroscopy. Furthermore, Current Density–Voltage (J-V) characteristics, Electrochemical Impedance Spectroscopy (EIS), and Incident Photon-to-Current Efficiency (IPCE) analyses are conducted to provide a comprehensive understanding of the kinetics governing the enhanced photovoltaic properties of the ZrO_2_ modified cells. To rigorously substantiate the empirical observations and convince the broader materials science community of the viability of an insulating material as an EBL, this study transitions from bulk conductivity assumptions to interfacial thin-film physics. By integrating mathematical modeling strategies—specifically quantum tunneling probability calculations and recombination kinetics models solved via MATLAB R2026B—we establish a robust theoretical foundation for the charge transport mechanisms. Ultimately, this dual empirical-theoretical approach not only elucidates the exact physical barrier effects of the ZrO_2_ layer but also introduces modern machine learning concepts as viable tools for future EBL optimization.

## 2. Materials and Methods

### 2.1. Production of Blocking Layers

The fabrication of the blocking layers was carried out via a hydrothermal methodology. Initially, commercial fluorine-doped tin oxide (FTO) substrates (TEC15, Pilkington, Ormskirk, UK) underwent a sequential ultrasonic cleaning process utilizing a detergent solution, deionized water, acetone, and isopropanol, with each step lasting 15 min. The substrates were subsequently dried under a steady stream of nitrogen gas. Prior to the fabrication of the TiO_2_ photoanode, electron blocking layers were deposited onto the conductive surface of the FTO glass. A TiO_2_ blocking layer was synthesized via a TiCl_4_ hydrothermal treatment [13]. Alternatively, the ZrO_2_ blocking layer was deposited by immersing the FTO substrates in a 5 mM solution of zirconium (IV) n-propoxide (Sigma-Aldrich, Darmstadt, Germany) dissolved in ethanol (Sigma-Aldrich Germany). This hydrothermal reaction was conducted in a Teflon-lined autoclave at 80 °C for 1.5 h, followed by an annealing process at 500 °C for 2 h.

### 2.2. DSSC Fabrication

The photoanodes were fabricated by depositing a TiO_2_ paste (Dyesol DSL-90T Australia) onto the bare FTO, TiO_2_/FTO, and ZrO_2_/FTO substrates using a screen-printing technique with a 90T mesh [16]. Surface profilometry measurements confirmed an active cell area of 0.25 cm^2^ and a mesoporous TiO_2_ film thickness of 14 µm. Following deposition, the photoanodes were dried at 120 °C for 30 min. A multi-step thermal annealing process was then performed under an ambient atmosphere at 325 °C for 5 min, 375 °C for 15 min, 450 °C for 15 min, and finally 500 °C for 15 min. Upon cooling, the annealed photoanodes were sensitized by immersion in a 0.5 mM N719 dye solution (Solaronix, Aubonne, Switzerland) at room temperature for 24 h in a dark environment. The counter electrodes were prepared utilizing a 5 mM ethanolic solution of hexa-chloro-platinic acid hexahydrate (H_2_PtCl_6_.6H_2_O). A droplet of this precursor solution was spin-coated onto the FTO substrate, followed by thermal treatment at 400 °C for 15 min to facilitate platinum deposition. To assemble the devices, the photoanode and the platinum counter electrode were sandwiched and sealed using a 25 μm thick Surlyn thermoplastic gasket (Dow-Türkiye, İstanbul, Turkey) at 100 °C. A liquid redox electrolyte (Solaronix AN50, Aubonne, Switzerland) was subsequently injected into the internal cavity via pre-drilled holes in the counter electrode. These injection ports were then hermetically sealed with additional Surlyn film, and the completed DSSCs were immediately subjected to photovoltaic characterization [17]. To ensure the statistical reliability and reproducibility of the results, five independent devices (N = 5) were fabricated and tested for each electrode configuration.

### 2.3. Mathematical Modeling Strategy

**Figure 2:**
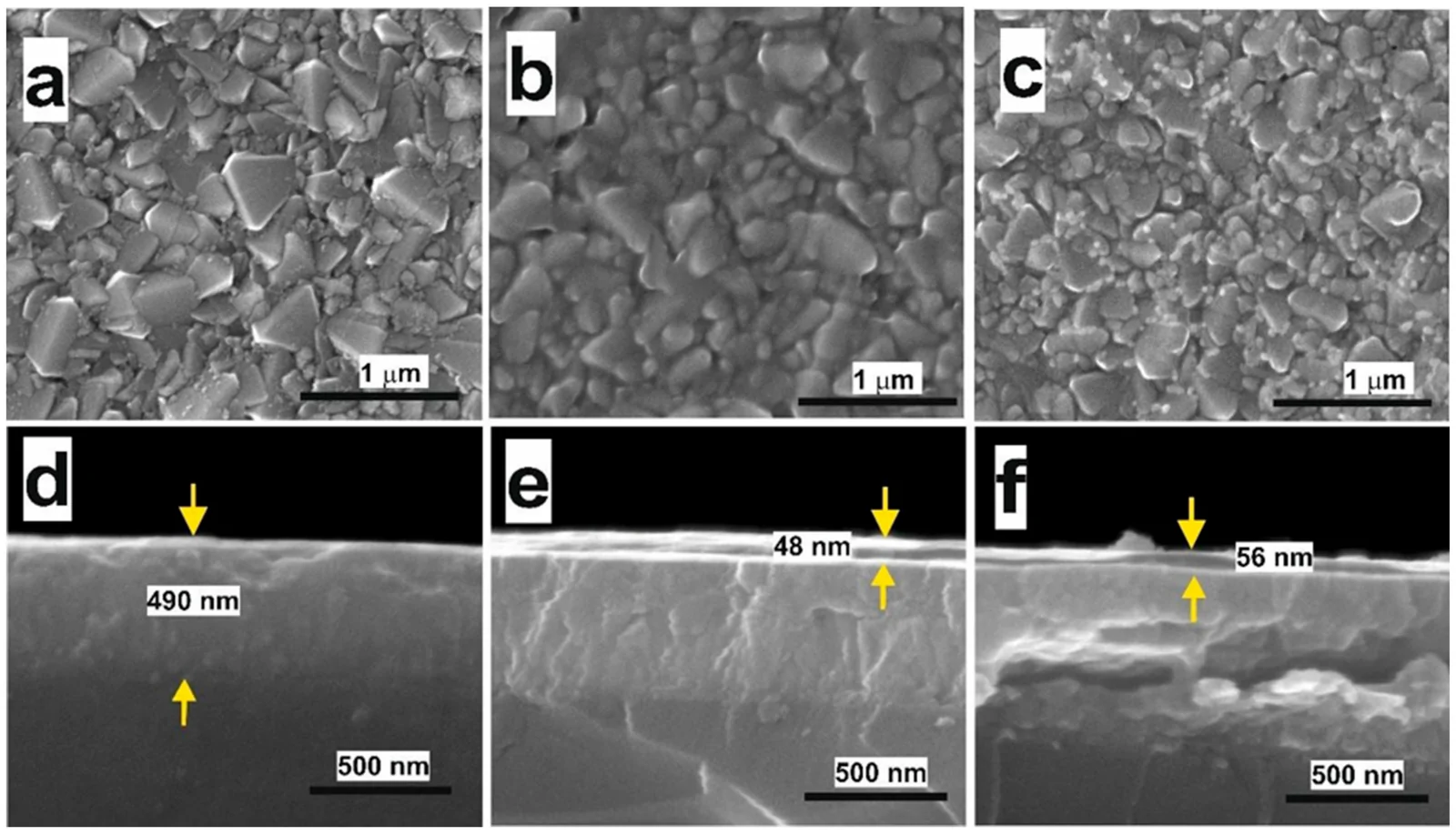
Top-view FE-SEM images of bare FTO (**a**), ZrO_2_/FTO (**b**), and TiO_2_/FTO (**c**); cross-sectional FE-SEM images of bare FTO (**d**), ZrO_2_/FTO (**e**), and TiO_2_/FTO (**f**). The thickness of the FTO layers was about 490 nm, and those of ZrO_2_ and TiO_2_ were about 48 and 56 nm, respectively.

To predict charge transport and theoretically validate the suppression of interfacial recombination, a quantum tunneling model and recombination kinetics equations were formulated utilizing MATLAB scripts. Since bulk ZrO_2_ is fundamentally an insulator, electrons injected from the dye into the TiO_2_ network must reach the FTO without recombining with the I^3−^ ions in the electrolyte. The probability of electron tunneling, *T*(*E*), through the dielectric ZrO_2_ layer is mathematically expressed as [18]:

(1) $$T\left(E\right)\approx \exp\left(-\frac{4\pi d}{h}\sqrt{2m^{*}\left(V-E\right)}\right)$$

where *d* is the barrier thickness 48 nm (in Figure 2e), *h* is Planck’s constant, m* is the effective mass of the electron, and (V−E) is the potential barrier height. Concurrently, to demonstrate how the ZrO_2_ layer minimizes electron leakage, the charge conservation and continuity equation for electrons in the mesoporous TiO_2_ network was modeled [19]

(2) $$\frac{\partial n}{\partial t}=D_{n}\frac{\partial^{2}n}{\partial x^{2}}-k_{rec}\left(n-n_{0}\right)+G$$

Here, *D_n_* represents the electron diffusion coefficient, *k_rec_* is the recombination rate constant, and *G* denotes the generation rate. In Equation (2), n represents the total dynamic electron concentration in the TiO_2_ conduction band under illumination, while $n_{0}$ denotes the thermal equilibrium electron density in the dark. The term ($n-n_{0}$) dictates the excess charge carriers driving the recombination kinetics. This differential model is utilized to fit the electrochemical impedance parameters, specifically the Z_1_ interface charge transport resistance and the Z_2_ interface resistance, to physically quantify the reduced leakage [20,21].

The physical parameters utilized in the 1D diffusion–recombination model were defined as follows: effective electron mass ($m^{*}$) for the ZrO_2_ layer = $0.1m_{e}$ (where $m_{e}$ is the rest mass of an electron), potential barrier height (V−E) representing the conduction band offset at the interface = 1.5 eV, electron diffusion coefficient ($D_{n}$) = $1\times 10^{-8}$ m^2^/s for TiO_2_ and $1\times 10^{-10}$ m^2^/s for ZrO_2_ recombination rate constant ($k_{rec}$) = 68.49 s^−1^ (derived from τ=0.0146 s), maximum generation rate (G) = $1\times 10^{21}$ m^−3^ s^−1^ with an exponential decay profile, and thermal equilibrium electron density ($n_{0}$) = $1\times 10^{16}$ m^−3^.

The one-dimensional continuity equation was solved numerically employing the explicit finite difference method (Forward Euler). The spatial domain was defined across the 14μm TiO_2_ network and the EBL interface using a uniform numerical grid (Nx = 400) with a spatial step size (Δx) of approximately 35.3 nm. To ensure numerical stability, the temporal evolution was simulated using a dynamically calculated time step ($\Delta t\approx 5.61\times 10^{-8}$ s) until a steady-state condition was achieved at t=0.05 s. Regarding the initial and boundary conditions, a uniform equilibrium concentration ($n\left(x,0\right)=n_{0}$) was set at t=0. A Dirichlet boundary condition ($n=n_{0}$) was applied at the left contact interface (x=0), while a zero-flux Neumann boundary condition (∂n/∂x=0) was established at the TiO_2_/electrolyte boundary.

## 3. Results

### 3.1. Morphological and Compositional Analyses of Coated and Uncoated FTO Surfaces

Field-emission scanning electron microscopy (FE-SEM) was utilized to evaluate the thickness and surface morphology of the respective films (Figure 2). The micrographs indicate a highly uniform deposition for the ZrO_2_ layer, whereas the TiO_2_ layer exhibited slight sedimentation phenomena intrinsic to the TiCl_4_ hydrothermal treatment. Cross-sectional analysis determined the bare FTO substrate thickness to be approximately 490 nm. The compact TiO_2_ and ZrO_2_ blocking layers exhibited thicknesses of 56 nm and 48 nm, respectively. Given the marginal dimensional variance between the two electron blocking layers (EBLs), their functional photovoltaic performances can be reliably compared [22].

X-ray photoelectron spectroscopy (XPS) is a highly sensitive surface analytical technique widely employed to investigate the dispersion, stoichiometric composition, and interfacial interactions of metal oxide films on conductive substrates [23,24]. Consequently, XPS was performed to confirm the structural integrity of the ZrO_2_ layer on the FTO.

**Figure 3:**
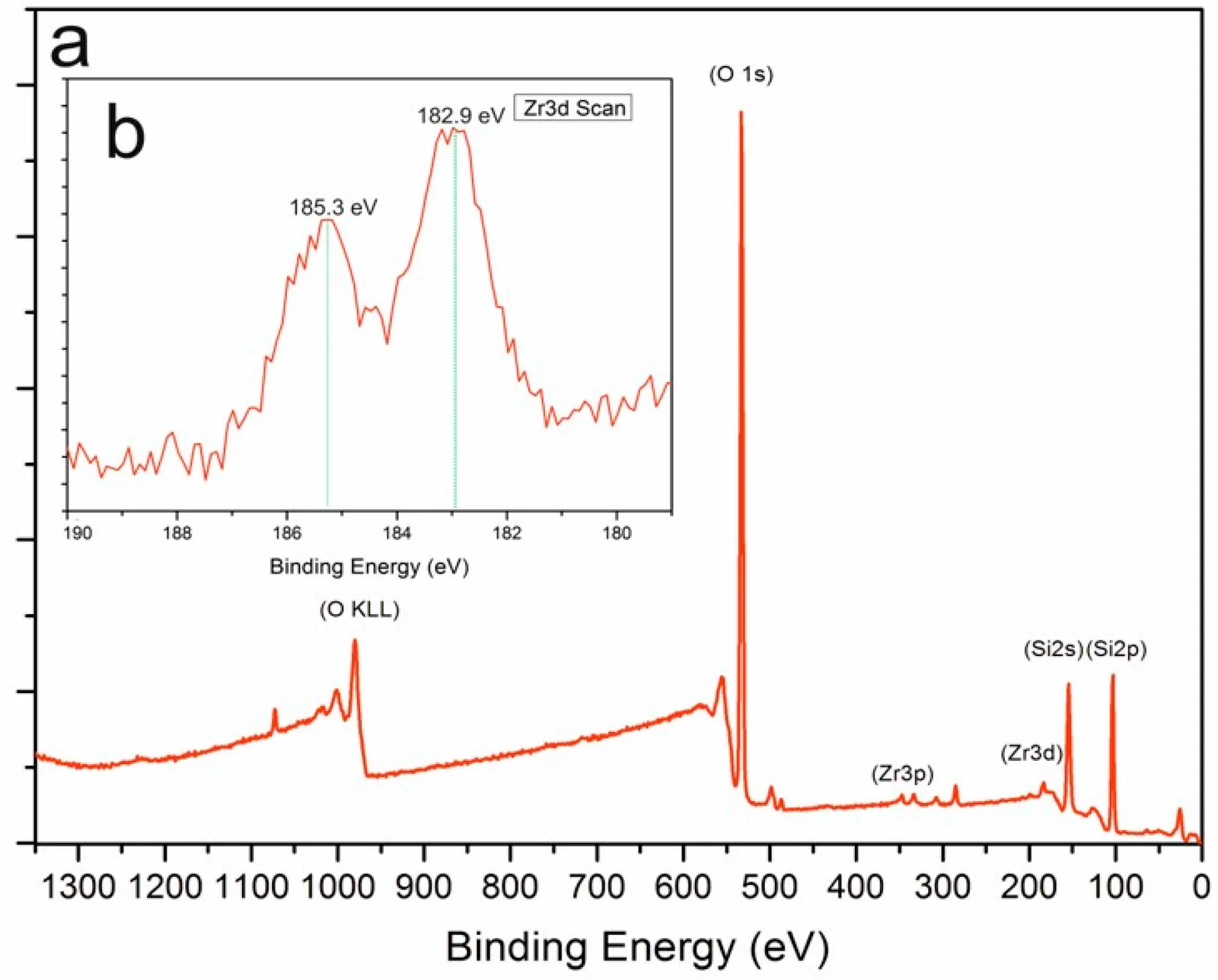
XPS survey spectrum with surface composition of ZrO_2_/FTO sample (**a**) and Zr 3d XPS spectra of ZrO_2_/FTO sample (**b**).

The survey spectrum of the ZrO_2_/FTO sample is depicted in Figure 3a. Furthermore, the high-resolution Zr 3d spectrum (Figure 3b) extracted from the 48 nm film reveals a characteristic 3d doublet splitting of 2.4 eV. The prominent Zr 3d_5/2_ peak is positioned at a binding energy of 182.9 eV, which is in excellent agreement with standard values for ZrO_2_ [25], thereby confirming the successful deposition of a pure ZrO_2_ blocking layer.

### 3.2. Optical Properties and UV-Vis Spectroscopy

**Figure 4:**
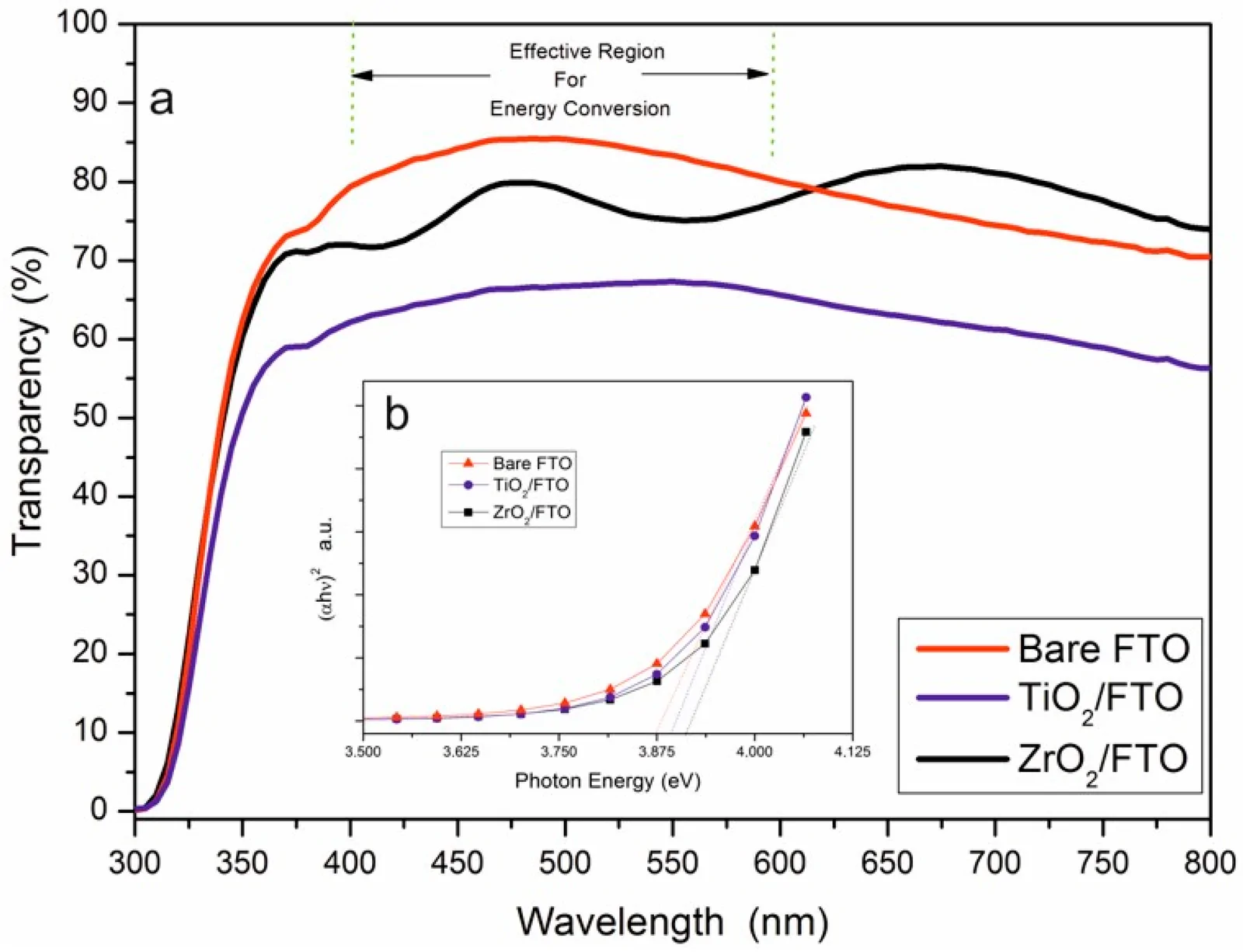
(**a**) UV-Vis transmittance spectra (300–800 nm) and (**b**) the corresponding Tauc plots showing the linear dependence of αhμ^2^ on photon energy (E, eV) for bare FTO, 56 nm TiO_2_/FTO, and 48 nm ZrO_2_/FTO thin films, The dashed lines indicate the corresponding energy values of 3.875 eV for bare FTO, 3.880 eV for TiO_2_/FTO, and 3.885 eV for ZrO2/FTO.

The optical transmittance of the bare and modified FTO substrates within the visible spectrum (300–800 nm) was evaluated using a UV-1900 UV-Vis spectrometer (Shimadzu UV-1900i Plus, Shimadzu Corporation, Kyoto, Japan) (Figure 4a). In the 300–620 nm region, both coated substrates exhibited slightly reduced transmittance relative to the bare FTO. However, at wavelengths exceeding 620 nm, the ZrO_2_/FTO configuration demonstrated superior optical transparency. This enhanced transmittance in the longer wavelength regime is highly advantageous, as it maximizes the photon flux reaching the mesoporous TiO_2_ dye interface, directly augmenting photoelectron generation. Although the FESEM analysis revealed comparable top-view surface morphologies for both electron blocking layers, the significant optical discrepancy observed in the UV-Vis spectra (>620 nm) is not attributed to a non-optimal morphology of the TiO_2_ reference layer. Instead, this difference originates from the intrinsic physical properties and distinct structural densities resulting from their respective synthesis routes. The TiCl_4_ hydrolysis method typically yields a denser TiO_2_ phase with a higher refractive index, which can induce slight parasitic optical scattering and thin-film interference at the FTO interface. In contrast, the hydrothermally deposited ultra-thin ZrO_2_ layer possesses a different intrinsic refractive index and structural density that effectively minimizes internal reflection and scattering in the longer wavelength regime, thereby preserving superior optical transparency. The direct optical band gaps of the films were determined via Tauc plot extrapolations from the low-wavelength transmission data (Figure 4b).

The calculated optical band gaps were 3.875 eV for bare FTO, 3.880 eV for TiO_2_/FTO, and 3.885 eV for ZrO_2_/FTO. A distinct blue shift in the UV-absorption edge was observed for the ZrO_2_-modified electrode. It should be noted that these values were calculated directly from the transparency-wavelength graphs. Since the deposited TiO_2_ and ZrO_2_ layers are ultra-thin (48–56 nm) compared to bulk materials, these data represent the effective optical thresholds of the composite FTO/EBL stack rather than pristine bulk band gaps. Therefore, these calculated values are highly accurate and fall well within acceptable limits for such specific nanoscale heterostructures.

This widened band gap limits parasitic light absorption by the EBL itself, allowing a greater proportion of the solar spectrum to penetrate into the active layer [26]. According to the Burstein–Moss effect, this blue shift indicates a displacement of the surface Fermi level toward higher energy states due to the occupation of lower conduction band levels [27,28].

### 3.3. Photovoltaic Performance (J-V Characteristics)

**Figure 5:**
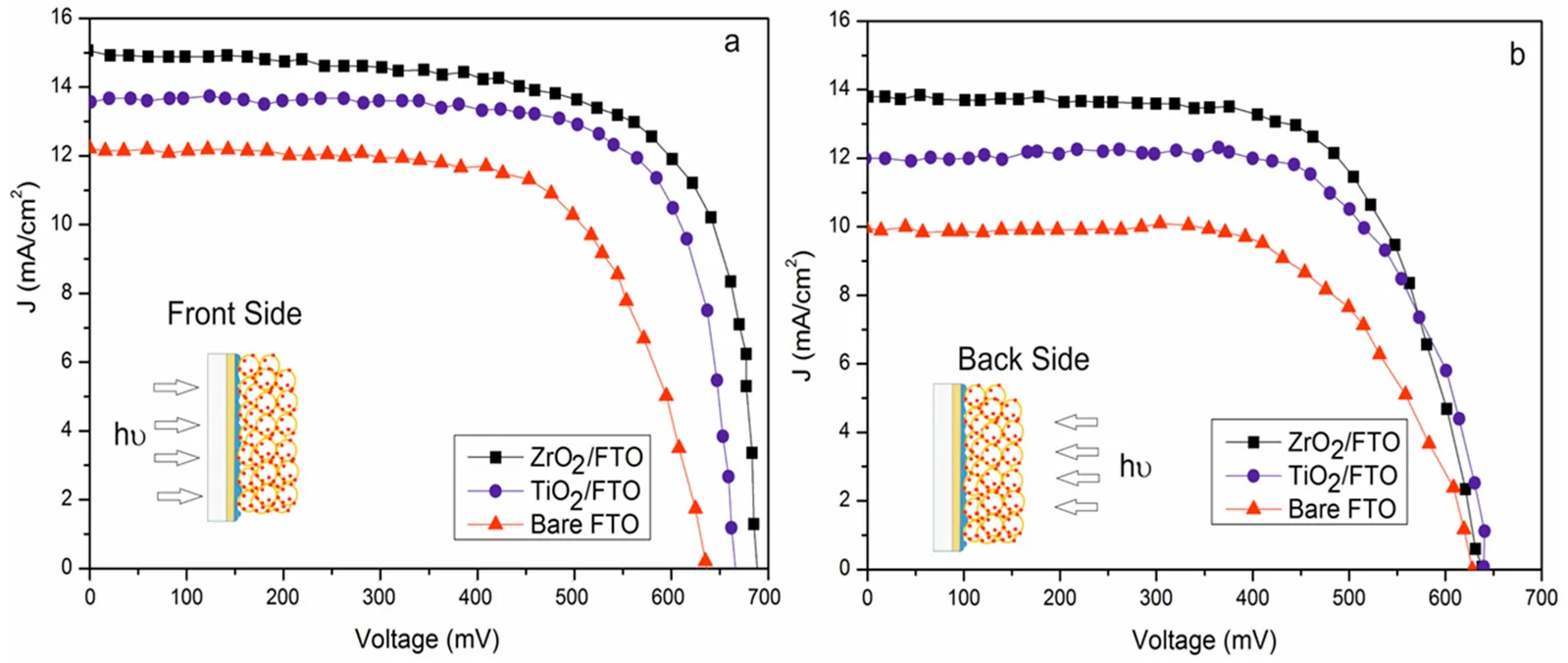
J-V curves of DSSCs employing bare FTO, ZrO_2_/FTO and TiO_2_/FTO layers electrodes with front (**a**) and backside (**b**) illumination (AM 1.5, 100 mW/cm^2^). The arrows indicate the direction of irradiation.

Photocurrent density–voltage (J-V) profiles of the fabricated DSSCs were acquired under simulated AM 1.5 illumination (100 mW/cm^2^) (Figure 5). Table 1 summarizes the corresponding photovoltaic parameters under both front and back illumination configurations. The data presented in Table 1 correspond to the mean values and standard deviations derived from the five independent samples, whereas the J-V curves displayed in Figure 5 illustrate the performance of the representative champion cells for each configuration. The integration of the ZrO_2_ blocking layer resulted in a remarkable 43.9% enhancement in overall power conversion efficiency (η) under front illumination. For context, conventional compact layer depositions typically yield efficiency improvements ranging from 30% to 53% [29,30,31]. The concurrent increases in short-circuit current density (Jsc), open-circuit voltage (Voc), and η for the modified cells suggest the effective suppression of interfacial charge recombination.

Specifically, the ZrO_2_-based DSSC achieved an η of 6.77%, outperforming the TiO_2_-based cell (5.72%) and the bare FTO reference (4.71%). Interestingly, the beneficial effects of the ZrO_2_ compact layer extended to backside illumination. Under this configuration, the Jsc of the ZrO_2_/FTO cell decreased by only 8.79%, whereas the bare FTO and TiO_2_/FTO cells experienced severe reductions of 11.49% and 16.46%, respectively. This superior retention of photocurrent can be attributed directly to the higher optical transmittance and the aforementioned Fermi level shift induced by the ZrO_2_ layer. Furthermore, the Voc improved from 640 mV (bare FTO) to 660 mV (TiO_2_) and 680 mV (ZrO_2_), underscoring the efficacy of ZrO_2_ in mitigating back-electron transfer dynamics. This upward shift in the effective surface Fermi level at the FTO/ZrO_2_ interface is fundamentally responsible for the enhanced charge extraction, which is clearly corroborated by the substantial increase in photovoltaic parameters observed under both front and backside illumination modes.

### 3.4. Incident Photon-to-Current Conversion Efficiency (IPCE)

**Figure 6:**
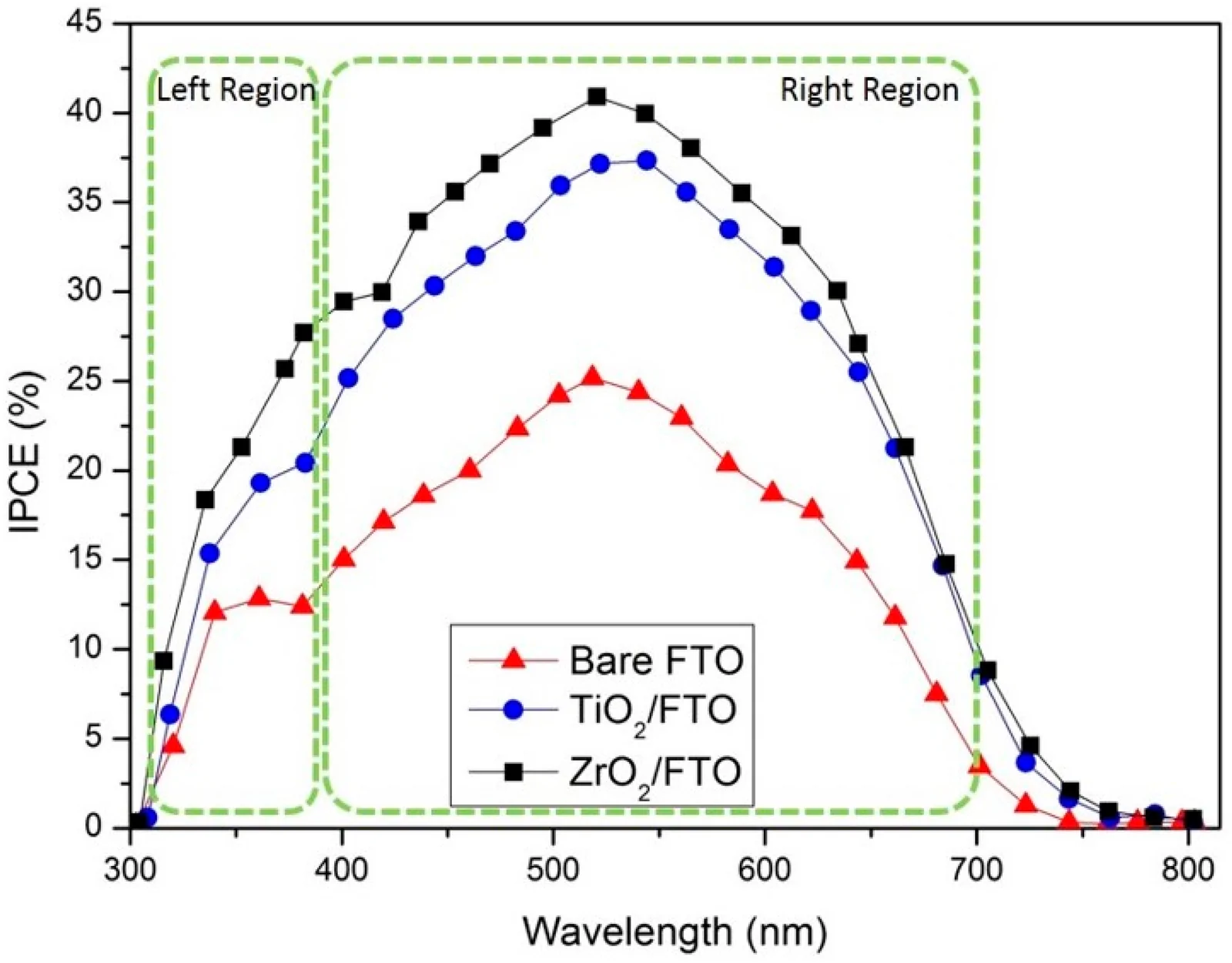
IPCE (Incident Photon-to-Current Efficiency) spectra for the DSSCs with and without blocking layers.

The IPCE spectra (Figure 6) which mathematically represent the External Quantum Efficiency (EQE) of the solar cells, elucidate the quantum efficiency of the devices and strongly correlate with the optical transmittance behavior. In the short-wavelength regime (below 390 nm), a minor peak near 370 nm is discernible for the TiO_2_-coated FTO, corresponding to the intrinsic bandgap absorption of the TiO_2_ nanoparticles [32]. Due to the lower molar extinction coefficient here, the dye sensitization effect is minimal. Conversely, the N719 dye dictates the spectral response in the visible region [33]. Notably, the ZrO_2_/FTO cell exhibited a distinct spectral peak at 420 nm, representing a beneficial red-shift compared to the bare FTO response, which translates to enhanced light harvesting [30]. The primary sensitization peak for N719 was prominently observed at 550 nm [34]. The device utilizing the ZrO_2_ EBL achieved a peak IPCE of 39%, significantly surpassing the 23% yielded by the bare FTO cell. Overall, the implementation of the ZrO_2_ layer resulted in a 69% relative enhancement in total IPCE, confirming its superior photon-management capabilities. Crucially, the substantial increase in the integrated area under this IPCE (EQE) spectral curve provides direct, independent physical evidence for the enhanced short-circuit current density (Jsc) observed in the J-V measurements (Table 1). This strict mathematical correlation conclusively validates the superior charge collection efficiency facilitated by the ZrO_2_ barrier.

### 3.5. Electrochemical Impedance Spectroscopy (EIS)

**Figure 7:**
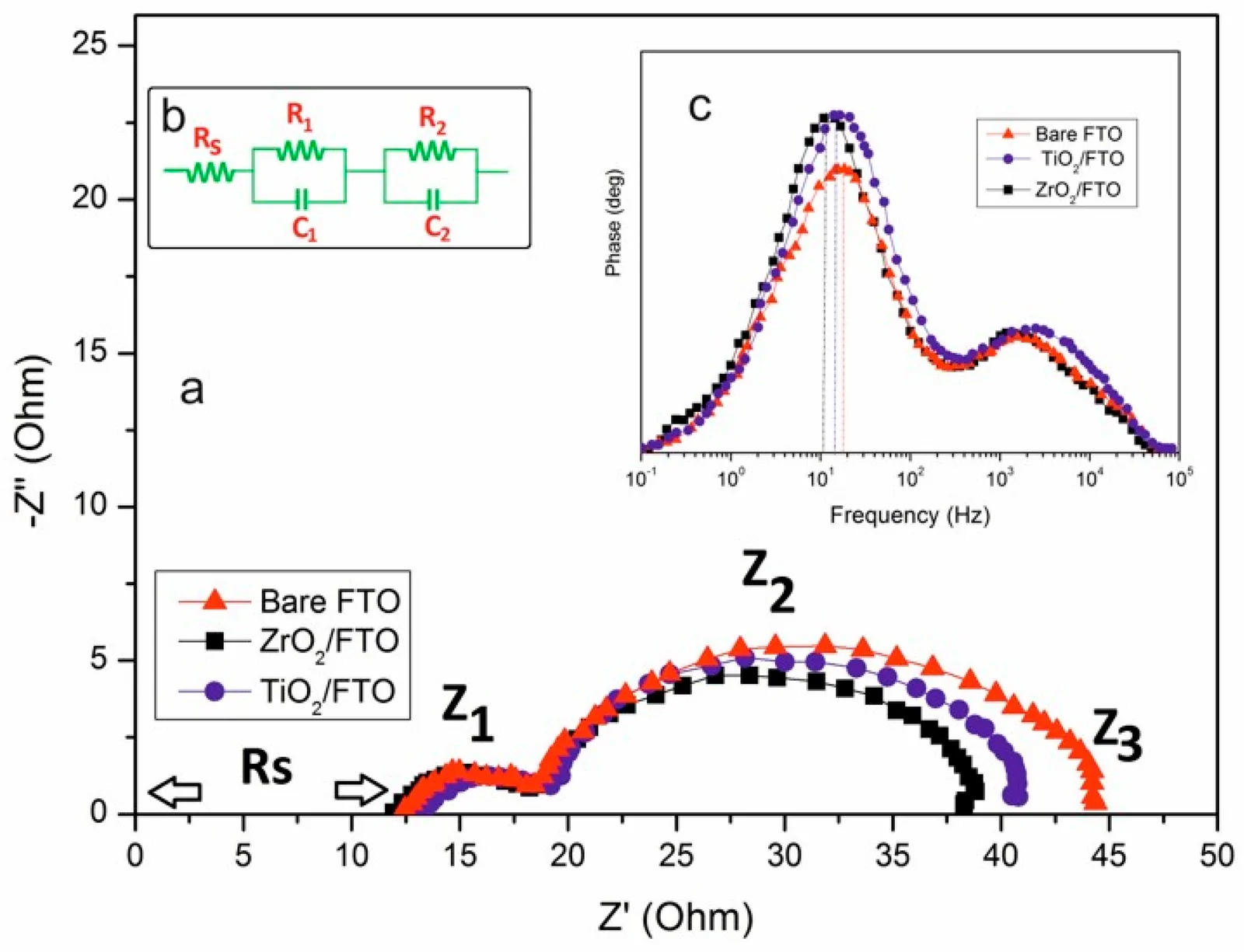
Representative Nyquist plots displaying impedance data taken at open-circuit potential (**a**), equivalent circuit of DSSC used for fitting impedance data (**b**), and Bode plots displaying impedance data (**c**). The characteristic fmax shifted from 18.49 Hz (bare FTO) to 16.43 Hz (TiO_2_/FTO) and down to 10.92 Hz for the ZrO_2_/FTO cell.

EIS was employed under one-sun, open-circuit conditions to interrogate the interfacial charge transport and recombination kinetics [35,36]. The resulting Nyquist plots (Figure 7a) display two characteristic semicircles across the 10^−1^ to 10^5^ Hz frequency range. The data were fitted using a standard equivalent circuit model (Figure 7b) [37].

The high-frequency element (Rs) represents the sheet resistance of the FTO substrate. The first semicircle (Ω_1_) reflects the charge transfer resistance (R_1_) at the conducting substrate/blocking layer/TiO_2_ and Pt/electrolyte interfaces. The mid-frequency semicircle (Ω_2_) corresponds to the recombination resistance (R_2_) at the TiO_2_/dye/electrolyte interface, while the low-frequency region (Ω_3_) relates to Nernstian diffusion within the electrolyte [38].

As detailed in Table 2, the ZrO_2_/FTO architecture exhibited the lowest R_1_ value, contributing positively to the macroscopic photovoltaic efficiency. More critically, the mid-frequency response provides the electron lifetime (*τ*), derived from the Bode phase peak frequency (*f_max_*) via the relation [39].

(3) τ=12πfmax

The characteristic fmax shifted from 18.49 Hz (bare FTO) to 16.43 Hz (TiO_2_/FTO) and down to 10.92 Hz for the ZrO_2_/FTO cell. This corresponds to a significantly extended electron lifetime (*τ* = 0.0146 s) for the ZrO_2_ modified device compared to the TiO_2_ reference (*τ* = 0.0097 s). The extended electron lifetime, coupled with lower charge transport resistance, definitively proves that the ZrO_2_ layer provides a robust kinetic barrier against interfacial recombination (electron leakage to the I^3−^ electrolyte).

### 3.6. Theoretical Validation and Optimization Pathways

The exceptional empirical performance of the ZrO_2_ EBL is fundamentally governed by nanoscale thin-film physics. At a measured thickness of 48 nm, the ZrO_2_ acts as a highly selective physical barrier. Based on the mathematical modeling strategy (Section 2.3), the low quantum tunneling probability *T*(*E*) across this 48 nm boundary mathematically confirms that undesirable electron leakage via backward transfer from the FTO to the electrolyte is heavily restricted. The continuity equation models align with the empirical EIS data, demonstrating that this restricted leakage leads directly to the extended electron lifetime (*τ* = 0.0146 s) and the lower charge transport resistance observed for the ZrO_2_/FTO configuration.

**Figure 8:**
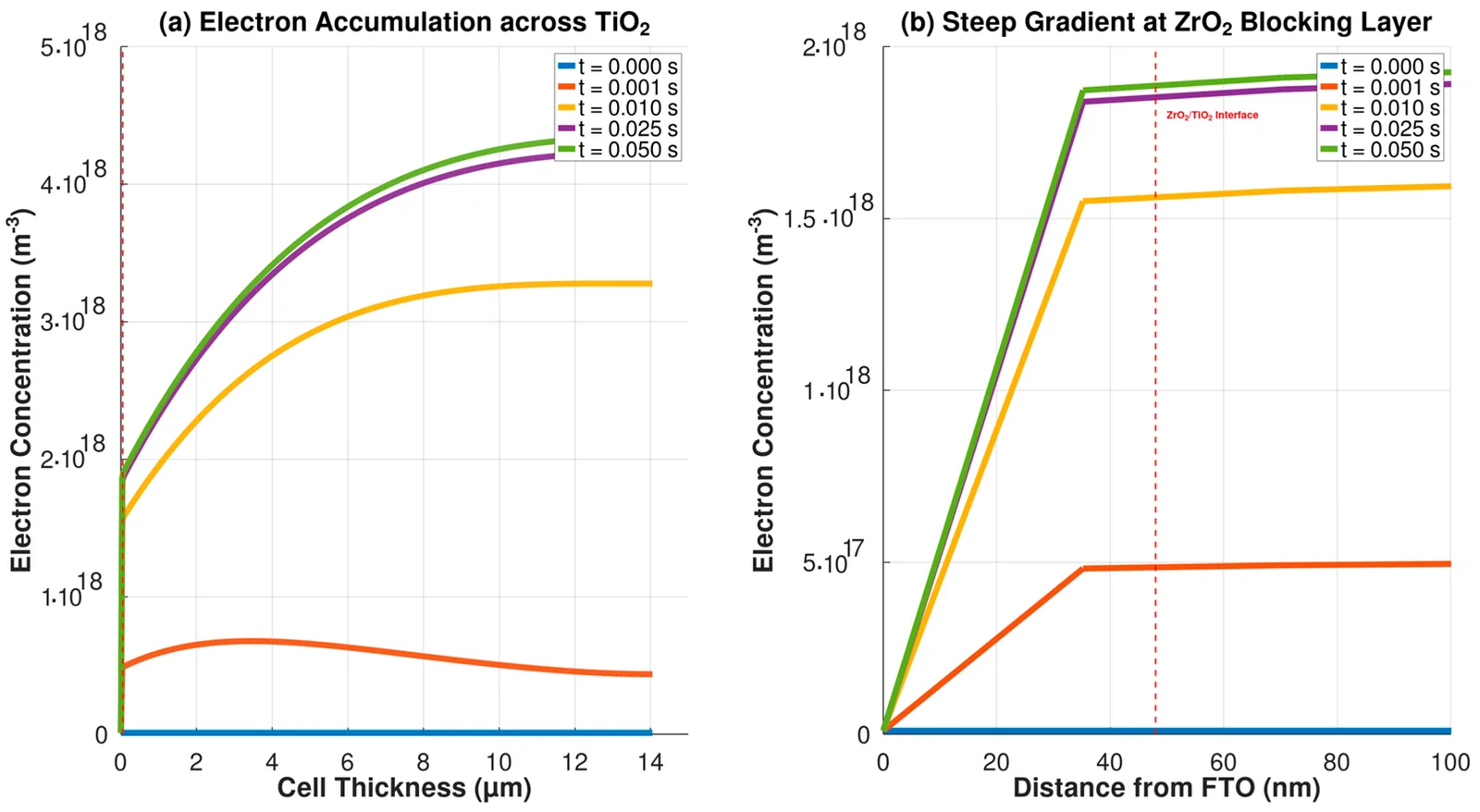
Time-dependent electron diffusion profiles simulated via the Crank–Nicolson method, illustrating (**a**) rapid electron accumulation across the 14 µm TiO_2_ network and (**b**) the steep concentration gradient established by the ZrO_2_ blocking layer at the interface.

**Figure 9:**
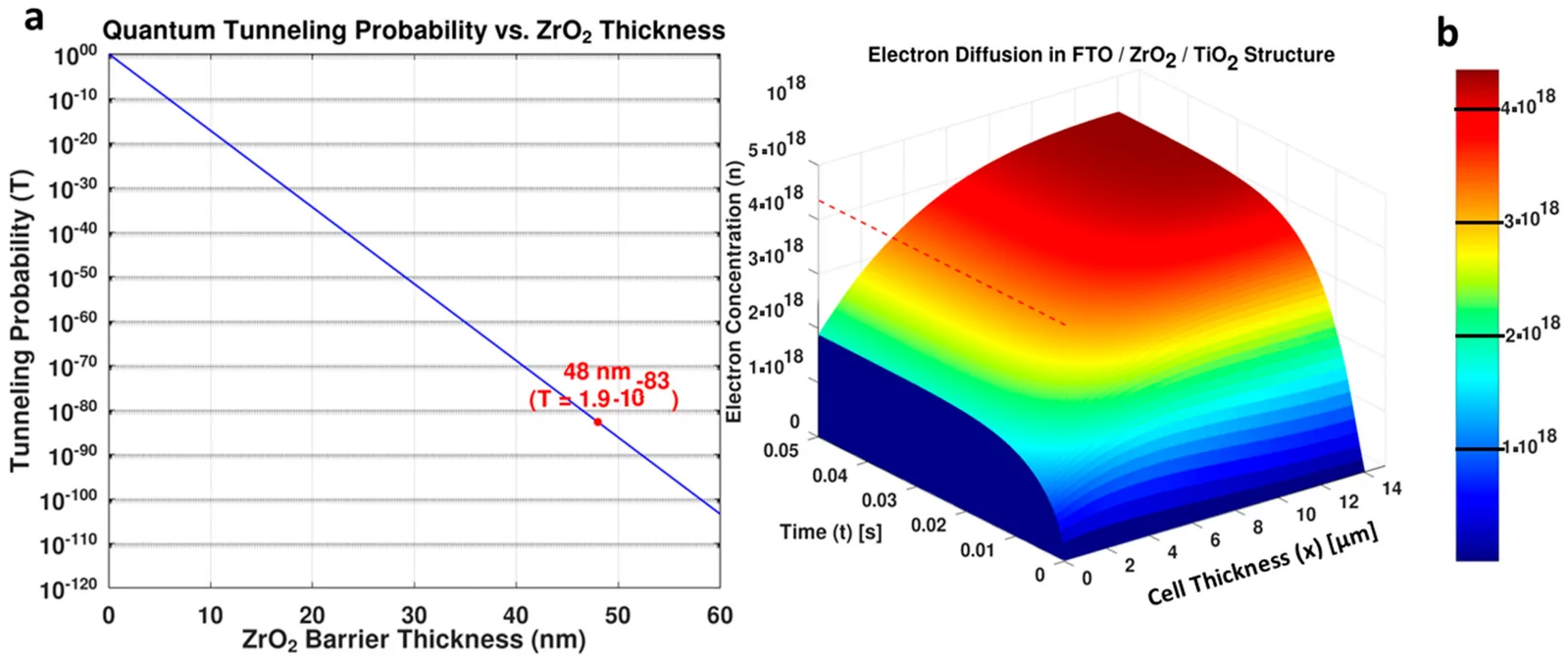
Mathematical simulations of the photoanode dynamics. (**a**) the WKB quantum tunneling probability as a function of ZrO_2_ barrier thickness, confirming that the experimental 48 nm layer physically prohibits reverse electron leakage (T = 1.9 × 10^−83^). (**b**) the 3D spatiotemporal electron diffusion and recombination profile, highlighting robust and continuous charge accumulation across the TiO_2_ film over time.

Time-dependent electron diffusion profiles across the photoanode stack simulated via the Crank–Nicolson method. Figure 8a illustrates the rapid accumulation of photogenerated electrons across the 14 µm mesoporous TiO_2_ network over time, while the magnified interfacial view in Figure 8b highlights the steep concentration gradient established by the ZrO_2_ blocking layer. This steep gradient visually confirms the layer’s role as a physical barrier, effectively restricting backward electron transfer to the FTO contact as the system reaches a steady state. To theoretically substantiate the barrier properties of the EBL, both the quantum tunneling probability and the spatiotemporal electron dynamics were mathematically simulated. As depicted in Figure 9a, the WKB approximation reveals that a 48 nm ZrO_2_ layer exhibits a near-zero tunneling probability (T ≈ 1.9 × 10^−83^). This infinitesimally small value mathematically validates that the ultra-thin ZrO_2_ film completely quenches quantum tunneling, effectively acting as an absolute physical barrier against reverse electron leakage to the electrolyte. Concurrently, the 3D surface profile derived from the continuity equation (Figure 9b) illustrates a rapid and robust accumulation of photogenerated electrons across the mesoporous TiO_2_ network. The sustained charge density over time further confirms that the ZrO_2_ compact layer successfully mitigates recombination losses, thereby maximizing the forward electron flux toward the FTO substrate.

Furthermore, the UV-Vis data reveals a blue shift in the optical band gap (from 3.875 eV to 3.885 eV), which, according to the Burstein–Moss effect, indicates that the surface Fermi level shifts toward a higher energy side, favorably aligning the energetics for maximum charge extraction. Looking forward, enriching these validated models with machine learning presents a powerful optimization pathway. Clustering algorithms, such as k-means or DBSCAN, could be applied to analyze the distinct 420 nm IPCE red-shift to mathematically isolate the specific wavelength regions where ZrO_2_ provides the highest quantum yield. Additionally, while the 48 nm thickness yields a 6.77% efficiency, future implementation of genetic algorithms could interpolate between physical constraints to predict the absolute “perfect” theoretical thickness (e.g., oscillating between 40 nm and 55 nm) to perfectly optimize the balance between optical transparency and electron blocking capability.

## 4. Conclusions

The pursuit of high-performance solar cells has driven extensive research into interfacial modifications, including solid additives [40] and specifically optimized TiO_2_ blocking layers [41]. However, this study introduces a fresh perspective to the literature by proving that substituting conventional materials with a wider-bandgap ZrO_2_ layer creates a far superior, exponentially stronger physical barrier against charge recombination.

In summary, the hydrothermal deposition of a 48 nm ZrO_2_ blocking layer yields profound enhancements in DSSC performance, establishing it as a highly viable alternative to conventional TiO_2_ EBLs. The ZrO_2_ layer optimally shifts the surface Fermi level to facilitate electron extraction, maintains excellent optical transparency. Electrically, it dramatically suppresses the back-electron transfer reaction, extending the electron lifetime and culminating in a 43.9% efficiency enhancement and a 69% IPCE improvement. The fundamental superiority of the ZrO_2_ blocking layer over conventional TiO_2_ is governed by its higher conduction band offset and the restriction of conductive trap states. These properties act synergistically to exponentially decrease the reverse tunneling probability, thereby establishing an optimal kinetic barrier against interfacial recombination without hindering forward electron extraction. Crucially, these empirical findings are rigorously validated by mathematical models; quantum tunneling probabilities and recombination kinetics confirm the ZrO_2_ layer’s function as a selective nanoscale physical barrier rather than a bulk insulator. Future research integrating machine learning algorithms with these mathematical models promises to unlock the absolute theoretical limits of blocking layer thicknesses for next-generation photovoltaics. Future research integrating predictive computational algorithms with these mathematical models promises to unlock the absolute theoretical limits of blocking layer thicknesses for next-generation photovoltaics.

## Figures and Tables

**Table 1:** Efficiency analysis of DSSCs employing bare FTO, ZrO_2_/FTO and TiO_2_/FTO layer electrodes with front and backside illumination.

	Backside Illumination				Front Illumination			
Sample	Voc (mV)	Jsc (mA/cm^2^)	FF (%)	η (%)	Voc (mV)	Jsc (mA/cm^2^)	FF (%)	η (%)
ZrO_2_/FTO	630 ± 9	13.80 ± 0.21	59.02 ± 0.89	5.13 ± 0.08	680 ± 18	15.13 ± 0.23	65.86 ± 0.99	6.77 ± 0.10
TiO_2_/FTO	640 ± 12	12.01 ± 0.22	55.01 ± 0.99	4.22 ± 0.08	660 ± 12	13.57 ± 0.24	63.88 ± 0.15	5.72 ± 0.10
Bare FTO	630 ± 10	10.09 ± 0.16	51.22 ± 0.82	3.25 ± 0.05	640 ± 10	12.08 ± 0.19	60.97 ± 0.98	4.71 ± 0.08

**Table 2:** Kinetic parameters of the DSSCs with and without blocking layers.

Sample	Rs (Ω)	R_1_ (Ω)	R_2_ (Ω)	*τ* (s)
ZrO_2_/FTO	11.9	5.01	16.03	0.0146
TiO_2_/FTO	12.3	5.24	22.96	0.0097
Bare FTO	12.5	6.02	26.44	0.0086

## Data Availability

The data presented in this study are openly available in Zenodo at https://doi.org/10.5281/zenodo.22862088.
